# Supplementary material for: Are carbon emissions trading and green financial instruments synergistic? -Comprehensive quantitative research based on content analysis
Source: PLoS One. 2024 Mar 7;19(3):e0298601. doi: 10.1371/journal.pone.0298601 (PMC10919601; doi:10.1371/journal.pone.0298601)
Supplement: S1 Data — (ZIP) [file pone.0298601.s001.zip › Data/地级市Z_F工作报告.zip!/║┌┴·╜¡/┤≤╟∞╩╨/└┤╫╘╣1⁄2╓┌║┼í╛┬φ┐╦╩2╛▌═°í┐_2018.docx]

各位代表：

　　现在，我代表市人民政府向大会报告工作，请予审议，并请市政协各位委员提出意见。

　　一、2017年工作回顾

　　2017年，我们深入学习贯彻党的十九大精神和习近平新时代中国特色社会主义思想，全面贯彻习近平总书记对我省重要讲话精神，深化落实省第十二次党代会精神和省委对大庆提出的“争当全国资源型城市转型发展排头兵”要求，按照市第九次党代会和市十届人大一次会议作出的安排部署，围绕转型发展“四重奏”、全面振兴“四条路”，聚精会神抓产业促升级，千方百计办实事惠民生，较好完成年初确定的各项目标任务，在新的起点上开创了经济社会发展新局面。

　　——经济运行态势回升向好。中省直与地方协同发力，传统产业与新增长领域共同支撑，预计全市实现地区生产总值2680亿元、增长2.8%，逐步走出“U”型曲线低谷。非油经济增长6.3%，地方财政收入增长15.9%，固定资产投资增长5%，同比分别提高3.5个百分点、22.1个百分点和3个百分点，增幅均创近3年新高。新建续建投资500万元以上重点产业项目288个，投产达产130个。农业提质增效。对接市场、调整结构、优化供给，粮食总产108.5亿斤、增长22%。“两牛一猪三禽”工程稳步推进，中以两国合作建设、落户杜尔伯特县的奶牛示范牧场建成投用。创新应用网络众筹、集团定制等营销模式，带动实现农产品销售收入70亿元，“鲶鱼沟”品牌荣登全省优质农产品营销大赛榜首，“连环湖”成为全国最具影响力水产品企业品牌。工业整体回暖。大庆油田全面盈利，生产原油3400万吨、天然气40.1亿立方米。专班专人专责推进的“油头化尾”上升为省级战略，具有重大牵动作用的大庆石化公司结构调整转型升级项目整体推进、4个子项目完成基础工程，550万吨重油催化热裂解、11万吨聚丙烯扩建等重点项目开工建设，林源化工园区热电站项目获得核准。汽车产业骨架隆起，沃尔沃汽车下线5.3万辆、出口2.2万辆，实现产值130亿元。农牧产品精深加工势头看好，笨嘴食品、一口猪二期等项目建成投产，伊品玉米深加工项目完成核心设备采购和基础土建施工。规上工业增量提质三年行动计划启动实施，帮助86户工业企业解决6.5亿元贷款周转，净增规上企业56户，地方规上工业增长10%以上。服务业贡献加大。市级自建电商平台“大庆·优品”上线运营，萨尔图区、肇州县电商园晋升省级，全市电商企业发展到304家、交易额90亿元。成功举办斯诺克国锦赛等15项品牌赛事，央视播出时长超过40小时。全市银行贷款增量和增速跃升至全省第2位、贷款余额突破1000亿元，新增国家级文化产业试验园区1个、国家4A级景区1个、民办养老机构8所。电商、文体、金融、旅游、康老等业态竞相发展，社会消费品零售总额增长5.8%。“双创”势头强劲。发放贴息创业担保贷款4516.9万元，新登记市场主体4万户、日均110户，城镇新增就业3.77万人。“三百”科技型企业三年行动计划收官，新注册科技型企业3246家，累计新培育主营业务收入500万元以上科技型企业410家。

　　——城乡群众生活持续改善。突出抓重点、补短板、强弱项，财政资金重点保民生，民生支出占公共财政支出78.1%，城乡居民年人均可支配收入分别达到38730元和14810元，增长6.1%和6.5%，收入水平位于全省前列。年度精准脱贫任务全面完成。重点实施产业扶贫、金融扶贫、帮扶扶贫、健康扶贫，落实扶贫项目150个，向上争取扶贫再贷款12.4亿元，杜尔伯特县达到脱贫摘帽标准，全市31个贫困村、7039名贫困人口脱贫。社会保障水平明显提高。城乡综合救助体系更加完善，发放基本生活保障金和救助金3.8亿元。完成农村低保清理整顿，取消低保对象36770人。四县月最低工资标准上调240元、达到1270元，市区上调230元、达到1680元、在省内处于领先位置。城乡居民医疗保险实现制度整合，农村居民大病保险筹资标准、贫困人口基本医疗保险住院费用报销比例、城乡居民养老保险待遇和失业保险标准再次提高。公共服务更加均衡。肇州县、红岗区等“三县三区”义务教育均衡发展通过国家验收，学前教育三年普及率达到90%、全省领先，改造义务教育薄弱学校89所，大庆成为中国首个普思示范城市。医疗体制改革扎实推进，公立医院全部取消药品加成，大庆入围全国首批健康城市试点、跻身国家基层中医药先进市。文化惠民工程深入实施，市图书馆、博物馆晋升国家一级馆。“智慧大庆”建设深入开展，14个信息惠民项目建成上线。人居环境优化提升。改造老旧小区11个、棚户区2736户、农村泥草危房1.3万户，2.8万套历史遗留房屋未办证问题得到有效解决，扩面降标完成2900套公租房源配租。落实“水十条”、“气十条”、“土十条”，淘汰黄标车1.1万辆、燃煤小锅炉46台，截至12月20日空气质量优良天数310天。城市轻轨线网规划编制完成，改造城乡道路85条，优化公交线路11条。取消让杜路收费，降低了出行成本。

　　——体制机制活力加速释放。坚持由点及面、由浅入深、由内向外，全面深化改革扩大开放。重点改革措施落地见效。立足“两个不能降、两个要上升”，以油田公司为重点的驻庆央企“三供一业”及企业办社会职能分离移交稳步推进。中石油集团电能公司在庆成立，油田售电公司业务拓展到11个省份。市属国企参股的中热新能源开发公司成立，地方混改取得突破。撤销公安消防建制，资产和职能划归武警消防。放宽市区落户限制，在全省率先实现“户籍通办”。网络预约出租车新政出台落地。高新区行政审批局、综合执法局启动运行。盘活政府非办公类资产79处、46万平方米。省水利学校迁址大庆。农村土地确权登记完成外业实测，农村集体产权制度改革试点启动推进。河长制在全市266个河湖得到全面落实。对外交流合作取得积极进展。通达大庆的中俄原油管道二线工程全线贯通、俄气东线管道开工建设，大庆能源供给地、集散地战略地位提升。萨尔图机场新开航线3条，旅客吞吐量突破60万人次。大庆沃尔沃汽车通过中欧班列和远洋货轮销往30多个国家和地区，打通了横跨欧亚、联通陆海的外贸通道。全市招商引进项目316个、到位资金315亿元。进出口总额77亿美元、增长37%。城市综合影响力大幅增强。高水平承办央视中秋晚会、全球观众超过7.5亿人次，参加央视“魅力中国城”竞演入围18强，32个国家和地区的38个涉外团组来庆访问交流，城市关注度和美誉度空前提升。大庆再次获评全国文明城市、国家卫生城市。

　　——综合发展环境不断优化。突出尊商重企便民，从关键部位和重要环节发力，推动环境建设走在全省前列。政策支持力度进一步加大。用好国务院赋予大庆“两个优先”激励政策，争取重点项目39个、资金5.04亿元。推出促进产业项目发展的意见，8个方面27条政策措施落地。设立产业基金二期，为重点项目提供扶持资金8亿元。降低工业用电用气价格，企业减少成本支出1.85亿元。落实小型微利企业和高新技术企业税收优惠政策，减税7066万元。行政服务能力进一步增强。市级行政许可事项精简至237项，行政许可中介事项减少到88项。企业投资项目承诺制试点方案进入审批程序，11个事项列入承诺范围。市政务云平台上线运行，完成国家“一号申请、一窗受理、一网通办”试点，超过20%的事项全程网上流转，审批提速64.2%。社会治理水平进一步提升。严查严管成品油市场、道路交通、食品药品等重点领域。未发生重大安全事故。社会治安防控体系建设不断深化，九项重大公安改革有效实施，八类主要刑事案件下降18.5%。重点信访历史积案有效化解，民族宗教领域依法管理得到强化，社会保持和谐稳定。

　　——政府自身建设切实加强。立足想干事、能干事、干成事的逻辑起点，务实求实开展工作，行政效能持续提升。民主法治建设不断深化。重大行政决策社会听证、政府法律顾问等项制度有效落实，决策更加科学民主。完善市政府常务会议规章制度，规范公文批办运转流程，集中清理规范性文件，政务工作标准化程度大幅提高。严格执行市人大及其常委会各项决议决定，自觉接受市人大法律监督、工作监督和市政协民主监督，312件市人大代表建议、229件市政协委员提案全部办结。廉政勤政得到加强。“两学一做”学习教育扎实开展，政府机关作风大整顿持续推进，坚持正向激励与负向约束相结合，市公安局被省政府记集体一等功、被市政府记集体二等功，给予推动重大项目向上争取工作取得重要进展的15家单位通报表扬，查处违反“八项规定”、破坏发展环境案件237件。政民沟通渠道拓宽。网络问政平台、市长热线等载体畅达民情，“您的美好向往、我们的奋斗目标”主题活动征询民生意见3590件次，部分意见建议吸收转化为政府重点工作任务，市民群众热爱家乡、共建家园的主人翁责任感充分彰显。

　　各位代表！

　　大庆发展取得喜人成果，主要得益于省委、省政府和市委的坚强领导，得益于油化大企业、中省直单位、高校院所、驻庆部队以及社会各界的鼎力支持，得益于全市人民的共同奋斗。在此，我代表市人民政府，向各位代表、各位委员和广大干部群众，向所有关心支持大庆发展的同志们、朋友们，表示崇高的敬意和衷心的感谢！

　　大庆发展变化令人振奋，但也要稳不忘忧、好中知难。主要是发展不平衡不充分的问题突出，影响转型发展的“三大矛盾”和“三偏问题”尚未有效破解，地方工业规模不大、层次不高，接续产业支撑乏力；改革开放力度不够，创新创业氛围不浓，国有经济缺少活力，民营经济发育不充分，一些体制机制弊端仍未革除；县域经济还很薄弱，主导产业不突出，村集体经济缺乏积累，农村基础设施和公共服务存在短板，脱贫攻坚还有很多硬仗要打；个别政府部门和公务人员慢作为、不作为的现象仍然存在，作风建设和廉政建设任务依然艰巨。这些问题，必须近远结合、标本兼治，下最大决心、尽最大努力加以解决。

　　二、2018年工作安排

　　2018年，是贯彻党的十九大精神的开局之年，是改革开放40周年，是决胜全面建成小康社会、实施“十三五”规划承上启下的关键一年。党的十九大发出了决胜全面建成小康社会、夺取新时代中国特色社会主义伟大胜利、实现中华民族伟大复兴的中国梦、实现人民对美好生活的向往的伟大号召，绘就了“两个一百年”的宏伟蓝图，指引我们阔步前行；省委十二届二次全会全面开启了建设富强民主文明和谐美丽社会主义现代化新龙江新征程，特别是省委寄予大庆“干在实处、走在前列、多做贡献”的殷切厚望，激励我们勇争排头；市委九届二次全会作出了争当全国资源型城市转型发展排头兵的重大战略部署，统筹把握“五个新时期”历史方位、“三个城市”发展定位、“两个阶段”宏伟目标，落实争当“排头兵”的意见和2018年行动计划，推动“五个转型”，打好打赢“八个攻坚战”，鞭策我们奋发有为。

　　新的一年，政府工作总体思路是：全面贯彻党的十九大精神，奋力践行习近平新时代中国特色社会主义思想，深入贯彻落实中央经济工作会议精神，认真落实省第十二次党代会、省委十二届二次全会和市委九届二次全会精神，按照高质量发展要求，突出供给侧结构性改革主线，传承弘扬大庆精神铁人精神，牢牢把握“五个要发展”根本路径，着力重构产业、重组要素、重聚动能、重塑环境，走出一条经济结构更优、体制机制更活、内生动力更足、承载功能更强的发展新路子，坚决打好打赢“八个攻坚战”，推动经济、城市、生态、体制、社会治理全方位转型升级，努力开创争当全国资源型城市转型发展排头兵、全面建设社会主义现代化新大庆的新局面。主要预期目标是：地区生产总值增长3.5%、力争达到4%，地方公共财政预算收入增长6.7%，固定资产投资增长10%，社会消费品零售总额增长6%，城乡居民年人均可支配收入分别增长6.2%和6.6%。

　　做好新时期政府工作，必须始终坚持党的领导，牢固树立“四个意识”，坚决维护以习近平同志为核心的党中央权威，确保中央、省委和市委各项决策部署落地生根；必须始终坚持深化密切地企关系，充分尊重油化大企业的历史性和现实性贡献，全力支持油田振兴发展，服务油化职工队伍创业创新；必须始终坚持依靠各方面的智慧和力量，在推动地企融合的同时，搞好市校融合、军民融合、城乡融合、区域融合，大庆的事业凝聚合力一起干，群众的事情尊重群众意见办，各方携手共谋转型、共促振兴、共享成果；必须始终坚持尽力而为、量力而行，既考虑现实急需又兼顾财力状况，既不急功近利也不好高骛远，少花钱办更多更好的事，一件接着一件办，一任接着一任干；必须始终坚持高标准、严要求、快节奏、求实效，决不躺在过去的功劳簿上，敢跟强的比，敢与勇的争，敢向高的攀，“争一保二不做第三”，推动各项事业晋位赶超。

　　2018年，我们要重点抓好八个方面工作：

　　（一）以建设现代化经济体系为引领，切实推动经济转向高质量发展阶段。立足市情阶段性特征，促进经济发展质量变革、效率变革、动力变革，加快由能源主导的单一型经济向多元综合型经济转变。着力振兴实体经济。树牢“不唯GDP，但也不能不要GDP”的理念，既重经济质量效益也重总量增速，巩固油化“压舱石”地位，提升高新区、经开区、各县区贡献率，力争非油经济增长6.9%、地方经济增长6.8%。推动5个千亿级集群产业和5个超500亿级重点产业建设取得新进展，为2020年完成“一步走一级跳”目标夯实基础。一产抓融合、二产抓提升、三产抓拓展，从大庆有鲜明供给优势的领域入手，制定实施培育新增长领域行动计划。促进个转企、小升规、规改股，壮大民营企业阵容，力争非公经济增长9%以上。强化创新驱动引擎。积极参与哈大齐国家自主创新示范区、“中国制造2025”哈大齐国家级示范区建设，实质性运作中科院长春分院大庆产业育成中心。争创国家科技成果转移转化示范区，引进建设多元化区域化创投孵化中心，完善提升现有科技企业孵化器集成功能，转化科技成果120项以上。支持企业自主研发创新，培育一批创新型排头兵企业。推进国家技术标准创新基地加快落地，实施质量强市、品牌强市战略，力促地产特色知名品牌培育取得更大突破。提升金融支撑经济发展能力。实施“引金入庆”计划，争取引进华夏银行、国鹏投资基金等金融机构，丰富金融业态，保持信贷合理增速。扩大直接融资，坚持储备一批、培育一批、辅导一批、上市一批，新增主板上市、新三板挂牌和进入辅导期企业4家。新设“油头化尾”产业基金、汽车产业投资基金，助推实体产业项目加快成长。推动人力资源协同发展。建好职业教育“双元”试点，培养更多企业急需的技术技能人才。实施大庆企业家培育计划，提升企业家现代生产经营综合能力素质。开展“人才引进高校行”活动，引入高水平创新团队、优秀青年人才和产业领军人才。支持驻庆高校加强学科建设和基础研究，充分发挥智力和创新支撑作用。

　　（二）以延伸拓展产业链价值链为抓手，打造多元支撑的工业格局。围绕做好“三篇大文章”、抓好“五头五尾”，坚持产业项目建设、创新驱动和新增长领域培育有机结合，推动能源稳市、工业强市、油化兴市。深化地企融合发展。推动技术创新、招商引资、共建园区、混合所有制改革等领域的深层合作，支持油化关联配套企业在地方注册。合力完成驻庆央企“三供一业”及企业办社会职能分离移交，携手办好事业、催生产业。倾力服务老油田与新区块稳步开发，生产原油3210万吨、天然气40亿立方米。集群发展“油头化尾”产业。抓住中石油集团与我省深化战略对接机遇，坚持“五个一块”保油头，推动大庆石化公司结构调整转型升级8个子项目按计划实施，争取漠大线俄油加工指标落地，支持大庆中蓝石化公司稳定生产，推进550万吨重油催化热裂解项目15套装置及配套设施尽快建设、20万吨高密度聚乙烯项目主体装置试车，开工建设煤炭分质清洁利用化工板块煤制乙醇项目。围绕延长“三条路径”强化尾，加快推进大庆炼化公司30万吨烷基化等重点项目，整合驻庆央企现有化工原料，落实百万吨聚烯烃等项目投资主体并尽早开工。推进宏伟园区污水处理厂建设，增强园区承载能力。整体升级汽车制造产业。围绕增型上产、延链扩能、关联配套，大庆沃尔沃工厂整车生产达到6万辆，引建S60车型，跟进保险杠、排气系统等零部件项目。加快引进新能源整车生产项目，力促大庆汽车城全面投用运营。持续壮大农副产品加工产业。围绕“粮头食尾”、“农头工尾”，集群式引建龙头型加工企业，推动中药材保健品、高端分离蛋白、玉米燃料乙醇等项目开工，建好惠丰乳业工业园等项目，确保伊品玉米深加工项目一期建成投产。加快推进新材料新能源产业。建设缘通碳纤维中试生产线，促进佳昌晶能新一代化合物半导体产业园落地开工。积极谋划、科学论证，建设后端铝材精深加工项目。争取可再生能源综合应用示范区获得国家批复，加快资源换项目、换产业、换市场，提高太阳能、风能、生物质能等新能源开发利用水平，形成新的增长领域，补充提升能源产业。大力扶持电子信息制造产业。完成华泰40万片蓝宝石衬底基片项目厂房改造及部分设备订购，建成映奥新一代显示屏研究院试验生产线。全市新增规上企业46户以上，地方规上工业增长10%以上。

　　（三）以扩大优势产品供给为突破，力促现代服务业融合升级。顺应中高端消费强劲增长趋势，加快培育新模式、应用新技术、创造新供给。活跃电商经济。推动电商园区提档升级，从交易支付、仓储包装、售后服务等环节入手，完善市级自建电商平台功能。坚持“本地注册、在地统计、属地纳税、当地就业”，新培育一批具有带动作用的电商企业，力争电商交易额突破100亿元。做强冰雪经济。融入全省“一区四带”建设，推出冰雪运动赛事、冰雪体验娱乐等7大主题24项产品。支持黑鱼湖等景区开发冰雪项目，叫响“百湖冰雪·雪地温泉”特色品牌，加快发展冰雪旅游、冰雪文化、冰雪体育、冰雪装备。打造旅游经济。深度开发工业、湿地、温泉、民俗等多样性资源，谋划“工业学大庆”红色小镇项目，启动建设“乡愁慢生活”田园综合体。实施景区景点品质升级行动，完善提升配套功能，提档棚室采摘、特色坑烤等体验旅游产品，全市旅游总收入突破170亿元。抓好赛事经济。重点办好斯诺克国锦赛、全国女篮联赛等品牌赛事。高水平承办第十四届省运会，办好全国大学生、中学生田径锦标赛，促进赛事影响力转化为经济助推力。涵养平台经济。引建物联网、云服务、短租经济等共享平台型企业，发展新业态新商业模式。争创国家新型智慧城市试点，推动华为云计算数据中心二期建成投用，争取省工业云、省政务云落户大庆。

　　（四）以实施乡村振兴战略为牵动，加快农业农村现代化步伐。落实“产业兴旺、生态宜居、乡风文明、治理有效、生活富裕”总要求，统筹实施乡村振兴战略五年行动方案，推动大庆乡村现代化建设走在全省前列。建设投入上多元支撑、滚动覆盖。每年从肇州县、肇源县、林甸县、杜尔伯特县、大同区50个非城关镇中选取10个乡镇集中建设，市财政专项列支2亿元、“四县一区”匹配2亿元、社会资本和受益主体注入1亿元，重点推动乡镇强产业、补短板、提功能、优环境，压茬铺开连续干5年，力争全市所有乡镇旧貌换新颜。改革措施上及时跟进、综合配套。健全城乡融合发展体制机制，清除阻碍要素下乡各种障碍。推进农村土地“三权分置”，完成全市土地承包经营权确权登记颁证工作，建设市县乡三级产权交易服务平台。深化农村集体产权制度改革，完成农村集体资产清产核资。拓展“两权”抵押贷款范围，活化融资渠道。探索小农户与现代农业有机衔接的实践路径，鼓励发展多种形式适度规模经营。资源配置上注重均衡、缩小差距。实施“10项基础振兴工程”，改造农村义务教育薄弱学校30所、乡村道路171公里、泥草危房1万户，提升乡镇卫生院20个，建设文化广场50个、垃圾收集点100个，铺设宽带光纤2000公里。推进农厕改造试点，逐步让农民用上干净的厕所。支持探索秸秆能源化利用新途径，提高秸秆综合利用水平。产业发展上整体联动、体现特色。坚持提质导向，支持农业县区大上项目、上大项目，推动宏福现代农业小镇一期等项目建成投用，壮大两江水稻、食品加工、棚室果蔬、特色水产等立县立区产业，打造全省的经济强县强区。激活乡镇经济、村集体经济，培育崛起一批旅游村镇、电商村镇、工艺村镇、匠人村镇。引导更多资本、技术、人才等要素向农业农村流动，完善农业产业体系、生产体系和经营体系。增收能力上多措并举、广辟途径。支持县区优化财源结构，增强可持续发展能力。发展集生产和销售于一体的新型农业经营主体，培养更多的农业经纪人、创业带头人。实施营销富农计划，通过卖得好促进农民增收。组织推进上挂图作战、务求实效。落实市、县区、乡镇三级主体责任，分季分段分步有序实施。强化督办，年终考核评估，好的激励、差的问责，形成乡村振兴的生动局面。

　　（五）以满足人民对美好生活的向往为目标，让改革发展成果更多更公平惠及城乡群众。围绕“七有”目标，立足坚守底线、突出重点、引导预期，突出问题导向、周密谋划、用心操作，倾听一个个民生诉求，办好一件件民生实事，交上一份份民生答卷。打赢精准脱贫攻坚战。坚持既不降低标准，也不吊高胃口，瞄准特定贫困群众精准帮扶。市财政投入专项资金1亿元，重点用于改造贫困村屯道路等基础设施。主攻产业扶贫软肋，因村因户发展特色种养、光伏扶贫等增收项目，增强“造血”能力。用绣花功夫推动“志智双扶”，实施“一村一品”产业行动和贫困户“一技之长”培育计划，确保全市31个贫困村、11065名贫困人口脱真贫真脱贫，精准退出不返贫。优先办好人民满意教育。巩固提升义务教育均衡发展成果，确保林甸县通过国家评估验收。调整优化教师队伍，补充农村和特殊教育师资力量。稳妥推进中考配额和应对新高考招生制度改革，加强国际办学与交流。迁建铁人中学，新建实验中学学生活动中心，扩建三永学校，推动祥阁学校二部和六十九中二部投用。减轻中小学生课外负担，解决“大班额”等问题。继续深入实施大庆精神铁人精神进校园进课堂，强化学校内涵建设，扬长补短擦亮教育金色名片。整体推进健康大庆建设。抓好健康城市试点，深化医疗体制改革、公立医院综合配套改革，落实分级诊疗制度。实施基层千人培养计划，强化全科医生培养和乡村医生学历教育。严格执行医疗机构药品购销“两票制”，降低群众就医负担。推进市中心血站、疾控中心改扩建，完善市县两级危重孕产妇和新生儿救治中心功能。深入落实文化惠民工程。举办“百湖百团百戏”精品展演。开展“书香大庆·悦读人生”全民阅读活动，推进公共图书资源进社区、进医院、进商场。创新升级“大庆之冬”、“激情之夏”群众文化品牌。争创国家公共文化服务体系示范城市。健全完善社会保障体系。全面落实全民参保计划，实现法定人员应保尽保。推进城乡居民基本医疗保险制度“八统一”，加快县区及以下公立医院卫生机构、村卫生室门诊统筹。推行全省“9+1+N”综合救助体系，提高城乡低保标准、特困人员救助供养标准。激励大学生、新型职业农民、科研人员、油化央企职工队伍等重点群体创业，新培育市场主体5万户。发展“银发经济”，完善居家和社区养老服务体系，建设养老服务中心40个。

　　（六）以提升宜居宜业功能为重点，系统推进城市修补生态修复。坚持尊重规律、简约适度、绿色低碳的价值取向，里子面子共同打造，修出品质、修出品位、修出品格。建设城市公共信息标识导向系统。突出科学规范，结合地域特征和文化特色，系统规范街区巷道和建筑门牌。突出多杆合一，有机整合主城区路灯杆、交通设施杆以及街路名牌、旅游导向牌。突出加减并重，集中清理规范车站、机场、高空等公共场所广告和沿街牌匾，增建电子路标、智能站牌，打造现代美观的城市形象。增强城市基础承载功能。推进哈尔滨至松原、大庆至明水铁路项目规划建设，促进区域基础设施互联互通。开工建设西城客运枢纽，编制轨道交通一期工程可研报告，维修改造净园路等破损、低洼、狭窄路段，在新建及改扩建的城市干道建设硬隔离步行道和自行车道。建成餐厨废弃物处理厂、建筑垃圾消纳场，改造生活垃圾处理厂。综合治理青龙山油田热电厂贮灰池。提升城市规划建设管理水平。加紧编制新一轮城市总体规划，抓好全省空间规划编制试点，推进“多规合一”。积极稳健推动房地产业发展。区块统筹、职能归口、政企分开，健全城市管理属地属事责任机制。修缮重点区域老旧公园、破损楼体、残缺字标，利用城市边角地、畸零地及闲置空间配建小游园小绿园。增加公交卡办理网点，动态开行旅游公交专线。合理增建公共停车场、停车泊位。有效疏导解决重点路段交通拥堵。加强道路运输客运市场规范管理。实行最严格的生态保护。全面落实河长制湖长制，编制“一河一策”实施方案，开展清河清湖行动。综合治理明湖、兰德湖等湖泡，整治大龙虎泡水源地，实施龙凤湿地生态修复工程，推动与绥化市建立安肇新河流域联合监测机制。实施国土绿化行动，引进培育一批专门从事生态保护修复的专业化企业。继续整治燃煤小锅炉，推广清洁能源供暖，集中治理面源污染，持续打好蓝天、碧水、黑土保卫战。开展领导干部自然资源资产离任审计，促进资源资产节约集约利用。

　　（七）以聚合域内域外发展动能为导向，进一步深化改革扩大开放。系统协同推进各项改革，拓展提升对外发展空间，全面增强社会生产力和创造力。突出重点领域，推动体制机制优化调整。加快市属国资国企重组整合，建立现代企业制度，打造主业突出、竞争力强的市属国企骨干集群。理顺政企关系，搭建融资平台，支持有盈利能力的公益类市属国企自主经营、自负盈亏。推动粮食集团启动运营。深化司法体制综合配套改革。加快推进生产经营类事业单位改革。突出重点环节，推动资源要素合理配置。全面实施市场准入负面清单制度，抓好企业投资项目承诺制试点，破除歧视性限制和各种隐性障碍。深化要素配置市场化改革，处置“僵尸”企业，破除无效供给。引导社会资本参与公共资源经营开发，促进政府非办公类资产和国企非经营性资产向生产部门配置。组建国有资本投资公司，深化公共资源交易改革，提高资源使用效益。围绕边际油田开发、石化链条延伸、新能源利用等领域，推动民营资本参与国企混改。突出重点区域，力促外向型经济发展。加快融入哈长城市群，对接省会城市建设提档升级三年行动，与哈尔滨市联手打造一小时经济圈。拓展“飞地经济”模式，支持县区与高新区、经开区合建共用产业园区。抢抓黑粤对口合作机遇，推动与惠州市在炼化一体化、装备制造、现代农业、新兴产业、科技创新、旅游、人才等领域合作的重点项目落地实施，确保取得务实成果。积极稳妥推进综保区和国际航空口岸建设。实施“名企入庆、定向招商、暖企助力”行动，力争到位资金突破330亿元。

　　（八）以集众智汇众力为支撑，形成共建共治共享的社会治理新格局。坚持专项治理、系统治理、综合治理、依法治理、源头治理相结合，加强社会治理“七个体系”建设，提高治理的社会化、法治化、智能化、专业化水平。系统防范化解社会矛盾。严格执行重大决策社会稳定风险评估机制，从源头上防范和减少隐患问题。建设四级综治中心，健全完善“三调联动”机制，加强社区矫正和安置帮教人员服务管理。构建多层次网上信访平台，推动律师深度参与信访工作，依法处理历史积案和信访问题。推进专业化社工队伍建设，强化基层社区网格功能，实施志愿服务制度化建设工程，形成矛盾纠纷多元化解机制。全力维护社会安定和谐。严防严控“六大风险”，加快社会治安防控体系建设，依法打击和惩治各类违法犯罪行为，全面“扫黑除恶”，深化反恐防暴斗争，建设更高水平的平安大庆。实施“雪亮工程”，优化整合各类智能化信息化平台，推动公安信息技术系统、城市监控系统升级和装备建设。压实安全生产“六大责任”，常态开展“四不两直”检查，确保安全生产形势总体稳定。正确引导社会预期。深入践行社会主义核心价值观，开展“引领风尚·铸魂大庆”活动，巩固提升全国文明城市创建成果。规范市长热线、网络问政等平台“接、转、答、解”流程，及时回应解决群众诉求。从源头入手，切实加强食品安全工作。建设以“一网、三库、一平台”为主体的公共信用信息系统，打破信息孤岛，促进政务诚信、商务诚信、社会诚信和司法公信。

　　各位代表！

　　推动大庆实现新发展，政府系统必须脚踏实地，全力抓好各项工作推进落实，确保有头有尾、有板有眼、有成有果。我们要勤于学习勇于创新。围绕“既要政治过硬，也要本领高强”，切实增强“八个本领”，努力适应政府工作新需要。注重学习先进地区发展经验，不断提升市场化融资、现代营销、商务谈判等专业化能力。加快掀起“头脑风暴”，实行自我革命，努力破除一切影响制约发展的旧思想、旧观念。我们要持续转变工作作风。严格落实中央八项规定及实施细则，认真执行省委、省政府和市委各项规定，发挥领导干部的“头雁效应”，采取见人见事的过硬措施，纠正“四风”不止步、驰而不息扬正气，以重点问题解决带动整体工作推进。深化政府系统作风大整顿，整治消极懈怠不担当等“坏作风”。拿出“破冰”勇气优化办事流程，努力让企业和群众办事像“网购”一样方便。坚持“抓两头带中间”，健全工作激励和容错纠错机制，选树一批铁人式、工匠型公务员。强化重点工作督查，政府工作全程留痕、线上督办，对县区经济发展实施季度考核、公开通报，年终兑现奖惩。我们要坚持推进依法行政。统筹实施法治大庆、法治政府、法治社会建设，确保政府工作始终在法治轨道上运行。深度精简调整市级行政权力，建立“不见面审批”事项目录，实行工商登记注册全域通办。推进“双随机一公开”，提升执法检查规范性。自觉接受市人大法律监督、工作监督和市政协民主监督，主动接受市监察委依法监察，认真听取人大代表、政协委员、民主党派、工商联和无党派人士意见建议，支持工会、共青团、妇联等群团组织开展工作。我们要切实加强勤政廉政建设。扎实推进“两学一做”学习教育常态化制度化，开展“不忘初心、牢记使命”主题教育。严格执行《准则》和《条例》，认真落实党风廉政建设主体责任。推行无纸化办公、无纸化会议。加强审计监督、效能监察，持续削减“三公”经费。强化重点领域、关键环节和重要岗位监管，严格执行招投标制度，确保公共资源、资产、资金在阳光下运行。

　　各位代表！

　　习近平总书记强调，“历史只会眷顾坚定者、奋进者、搏击者，而不会等待犹豫者、懈怠者、畏难者”。迈入新时代、实现新发展、建设新大庆，必须恪尽职守，奋发有为。让我们在市委的坚强领导下，团结带领全市人民，发扬大庆精神铁人精神，勇于变革、勇于创新，永不僵化、永不停滞，为决胜全面建成小康社会、争当全国资源型城市转型发展排头兵而努力奋斗！
